# Nearly Complete Genome Sequence of *Lactobacillus plantarum* Strain NIZO2877

**DOI:** 10.1128/genomeA.01370-15

**Published:** 2015-11-25

**Authors:** Maria Elena Martino, Jumamurat R. Bayjanov, Pauline Joncour, Sandrine Hughes, Benjamin Gillet, Michiel Kleerebezem, Roland Siezen, Sacha A. F. T. van Hijum, François Leulier

**Affiliations:** a Institut de Génomique Fonctionnelle de Lyon (IGFL), École Normale Supérieure de Lyon, CNRS UMR, Université Claude Bernard Lyon 1, Lyon, France; b Center for Molecular and Biomolecular Informatics, Nijmegen, The Netherlands; c Host Microbe Interactomics Group, Wageningen University, Wageningen, The Netherlands; d Microbial Bioinformatics, Ede, The Netherlands; e NIZO food research, Ede, The Netherlands

## Abstract

*Lactobacillus plantarum* is a versatile bacterial species that is isolated mostly from foods. Here, we present the first genome sequence of *L. plantarum* strain NIZO2877 isolated from a hot dog in Vietnam. Its two contigs represent a nearly complete genome sequence.

## GENOME ANNOUNCEMENT

Lactic acid bacteria are Gram-positive bacteria that have extensively been used in the food industry (1). *Lactobacillus plantarum* is one of the most important and widespread members of the genus *Lactobacillus*, and it is mainly isolated from plant- and meat-derived foods. *L. plantarum* is also used as a probiotic because of its beneficial effects on human and animal health (2, 3). Here, we report the draft genome sequence of *L. plantarum* strain NIZO2877 isolated from a hot dog in Vietnam.

Total DNA from *L. plantarum* NIZO2877 was extracted and subjected to 150-bp paired-end sequencing using Illumina MiSeq technology (Illumina, San Diego, CA) at ProfileXpert (Lyon, France). A total of 1,820,816 reads were obtained and assembled using Ray (4) in 23 contigs. Gap closing was conducted using Sanger sequencing. Twenty-three pairs of primers were designed between contigs using the Primer3 software (5). The endpoint PCRs were conducted on a Veriti Applied Biosystems thermocycler (Life Technologies, Carlsbad, CA). Twenty-two gaps were closed, and the resulting assembly consisted of 2 chromosomal contigs (1,275,359 bp and 1,956,412 bp). The total genome length is 3,231,771 bp. The G+C content is 44.49%, which is consistent with the results reported for other *L. plantarum* genomes (44.5 to 44.7%). Annotation was performed using the RAST annotation server (6). A total of 3,165 protein-coding sequences (CDSs) were identified; defined biological functions were assigned to 2,424 CDSs. Six hundred seventy-five CDSs were homologous to sequences encoding hypothetical proteins in other organisms, and 66 CDSs did not have a database match. The genome contains 68 tRNA genes and 14 rRNA genes.

While exploring the specific features of *L. plantarum* NIZO2877, we found that it has a truncated *hepB2* gene (EC 2.5.1.30, heptaprenyl diphosphate synthase), which converts farnesyl diphosphate to heptaprenyl diphosphate in the vitamin K_2_ biosynthesis pathway. This feature is present in only one other *L. plantarum* strain, ATCC 14917 (7). However, the two strains bear a complete copy of the *hepB1* gene that has the same function as *hepB2*, hinting that the truncated copy of *hepB2* does not result in a loss of function.

The majority of *L. plantarum* genome sequences were derived from dairy product or plant isolates. The availability of the genome sequence of *L. plantarum* NIZO2877, isolated from a meat product, increases our knowledge of other food-derived *L. plantarum* strains and will assist in a better understanding of its ecology.

### Nucleotide sequence accession numbers.

This whole-genome shotgun project has been deposited at DDBJ/EMBL/GenBank under the accession no. LKHZ00000000. The version described in this paper is version LKHZ01000000.

## References

[1] Makarova K, Slesarev A, Wolf Y, Sorokin A, Mirkin B, Koonin E, Pavlov A, Pavlova N, Karamychev V, Polouchine N, Shakhova V, Grigoriev I, Lou Y, Rohksar D, Lucas S, Huang K, Goodstein DM, Hawkins T, Plengvidhya V, Welker D, Hughes J, Goh Y, Benson A, Baldwin K, Lee JH, Díaz-Muñiz I, Dosti B, Smeianov V, Wechter W, Barabote R, Lorca G, Altermann E, Barrangou R, Ganesan B, Xie Y, Rawsthorne H, Tamir D, Parker C, Breidt F, Broadbent J, Hutkins R, O’Sullivan D, Steele J, Unlu G, Saier M, Klaenhammer T, Richardson P, Kozyavkin S, Weimer B, Mills D 2006 Comparative genomics of the lactic acid bacteria. Proc Natl Acad Sci USA 103:15611–15616. doi:10.1073/pnas.0607117103. 17030793 PMC1622870

[2] Cebeci A, Gürakan C 2003 Properties of potential probiotic *Lactobacillus plantarum* strains. Food Microbiol 20:511–518. doi:10.1016/S0740-0020(02)00174-0.

[3] Siezen RJ, van Hylckama Vlieg JE 2011 Genomic diversity and versatility of *Lactobacillus plantarum*, a natural metabolic engineer. Microb Cell Fact 10(Suppl 1):S3. doi:10.1186/1475-2859-10-S1-S3. 21995294 PMC3271238

[4] Boisvert S, Raymond F, Godzaridis É, Laviolette F, Corbeil J 2012 Ray Meta: scalable *de novo* metagenome assembly and profiling. Genome Biol 13:R122. doi:10.1186/gb-2012-13-12-r122. 23259615 PMC4056372

[5] Untergrasser A, Cutcutache I, Koressaar T, Ye J, Faircloth BC, Remm M, Rozen SG 2012 Primer3—new capabilities and interfaces. Nucleic Acids Res 40:e115. 22730293 10.1093/nar/gks596 PMC3424584

[6] Aziz RK, Bartels D, Best AA, DeJongh M, Disz T, Edwards RA, Formsma K, Gerdes S, Glass EM, Kubal M, Meyer F, Olsen GJ, Olson R, Osterman AL, Overbeek RA, McNeil LK, Paarmann D, Paczian T, Parrello B, Pusch GD, Reich C, Stevens R, Vassieva O, Vonstein V, Wilke A, Zagnitko O 2008 The RAST server: Rapid Annotations using Subsystems Technology. BMC Genomics 9:75. doi:10.1186/1471-2164-9-75. 18261238 PMC2265698

[7] Tatusova T, Ciufo S, Fedorov B, O’Neill K, Tolstoy I 2014 RefSeq microbial genomes database: new representation and annotation strategy. Nucleic Acids Res 42:D553–D559. doi:10.1093/nar/gkt1274. 24316578 PMC3965038

